# What about lay counselors’ experiences of task-shifting mental health interventions? Example from a family-based intervention in Kenya

**DOI:** 10.1186/s13033-020-00343-0

**Published:** 2020-02-20

**Authors:** Jonathan T. Wall, Bonnie N. Kaiser, Elsa A. Friis-Healy, David Ayuku, Eve S. Puffer

**Affiliations:** 1 Duke University, Durham, NC USA; 2 University of California San Diego, La Jolla, CA USA; 3 Moi University College of Health Sciences, School of Medicine, Eldoret, Kenya

**Keywords:** Mental health, Lay counselors, Task shifting, Africa, Burnout, Motivation, Self-efficacy, Stress, Family therapy, Children

## Abstract

**Background:**

A key focus of health systems strengthening in low- and middle-income countries is increasing reach and access through task-shifting. As such models become more common, it is critical to understand the experiences of lay providers because they are on the forefront for delivering care services. A greater understanding would improve lay provider support and help them provide high-quality care. This is especially the case for those providing mental health services, as providing psychological care may pose unique stressors. We sought to understand experiences of lay counselors, focusing on identity, motivation, self-efficacy, stress, and burnout. The goal was to understand how taking on a new provider role influences their lives beyond simply assuming a new task, which would in turn help identify actionable steps to improve interventions with task-shifting components.

**Methods:**

Semi-structured interviews (n = 20) and focus group discussions (n = 3) were conducted with three lay counselor groups with varying levels of experience delivering a community-based family therapy intervention in Eldoret, Kenya. Thematic analysis was conducted, including intercoder reliability checks. A Stress Map was created to visualize stress profiles using free-listing and pile-sorting data collected during interviews and focus group discussions.

**Results:**

Counselors described high intrinsic motivation to become counselors and high self-efficacy after training. They reported positive experiences in the counselor role, with new skills improving their counseling and personal lives. As challenges arose, including client engagement difficulties and balancing many responsibilities, stress and burnout increased, dampening motivation and self-efficacy. In response, counselors described coping strategies, including seeking peer and supervisor support, that restored their motivation to persevere. At case completion, they again experienced high self-efficacy and a desire to continue.

**Conclusions:**

Findings informed suggestions for ways to incorporate support for lay providers into task-shifting interventions at initiation, during training, and throughout implementation. These include acknowledging and preparing counselors for challenges during training, increasing explicit attention to counselor stress in supervision, fostering peer support among lay providers, and ensuring a fair balance between workload and compensation. Improving and building an evidence base around practices for supporting lay providers will improve the effectiveness and sustainability of lay provider-delivered interventions.

## Background

A major issue facing health systems in low- and middle-income countries (LMICs) is improving healthcare accessibility resulting from health care professional shortages, especially for mental healthcare [1, 2]. Much work advocates for health systems strengthening through task-shifting, or task-sharing, by training non-specialists or non-professionals to provide services [3–5]. Most often, such programs employ community health workers (CHWs), more broadly referred to as lay providers, who deliver services ranging from HIV-Tuberculosis care and management [6] to mental healthcare, the topic of the current study [7, 8]. Task-shifting has become the de facto model of much mental healthcare delivery in low-resource settings globally due to the one-million-person shortage of mental health specialists [4, 9].

Despite the many benefits of task-shifting for increasing healthcare accessibility, a growing body of research points to challenges lay providers face. A key challenge is socioeconomic inequities between lay providers and employed health professionals. This reality is largely driven by interventionists pushing an ethic of volunteerism for lay provider programs [10, 11]. Some policymakers and community program leaders claim that lay providers are “priceless” and might lose intrinsic motivation to fulfill their responsibilities if paid [12, 13]. Additionally, many programs emphasize their cost-effectiveness in increasing healthcare accessibility, which is premised on not paying lay providers [14, 15]. This is a topic of ongoing debate in the field and has important implications for intervention delivery [11, 16].

Beyond economic impacts, the four most common areas of concern regarding lay provider experiences are motivation, self-efficacy, stress, and burnout (Table 1), any of which can contribute to poor retention among lay providers [17]. These are concerning from an individual well-being perspective and because they might reduce quality and effectiveness of interventions. These outcomes are even more concerning among lay counselors, a specific type of lay provider focused on mental healthcare, which often requires more time commitment and raises the risk for unique stressors, including compassion fatigue. One proposed root cause of these problems is that lay providers are sometimes treated as a “means to an end,” rather than as individuals who may need support to optimize their services [18–20]. Therefore, some researchers propose fostering a humanistic view of lay providers, encouraging global health actors not to approach lay providers as technocratic solutions, but as people with unique skills, desires, and perspectives [21].

**Table 1:** Study domains of interest

Motivation	Self-efficacy	Stress	Burnout
Intrinsic and extrinsic forces, beliefs, and ideals that incline an individual to pursue and maintain their position as a lay counselor [22]	An individual’s perceptions and assessments of their capability to be competent and effective in their counseling role [23]	The physical, psychological, emotional, and social consequences that affect a lay counselor due to trying to fulfill the duties and responsibilities expected of them by their clients, supervisors, and communities	Combination of feelings of work-related exhaustion, cynicism, and inefficacy due to the daily routine and duties required for a counselor, which can manifest in physical and behavioral changes [24, 25]

Although past studies have pointed toward workload and socioeconomic inequities as driving problems of de-motivation and burnout among lay providers, another possible cause is the stress of taking on the new role itself [26]. Role identity theory provides a useful framing for exploring shifts in identity that lay providers may experience because of taking on a new role, and how these shifts relate to stress or resilience. The theory posits that all people have multiple, hierarchically arranged identities and roles that motivate behaviors [27]. The roles are thought to be intimately related to each other, often influencing how other roles are performed and shaping the personal meaning of individual identities [28]. As individuals learn new skills and interact socially, they constantly acquire new roles that add to their “role set,” like adding tools to a toolkit. Role shifts are then defined as change or reshaping of a role set because of new relationship interactions, social positions, or duties. For lay providers, such shifts in social roles occur after they receive training and take on new health worker roles. The current study applies this theory with the goal of generating potential avenues for better lay provider support.

### Study aims

We used a humanistic approach incorporating role identity theory to examine experiences of lay counselors trained in a family therapy intervention in Eldoret, Kenya. We aimed to describe how lay counselors experience role shifts and associated outcomes, such as motivation and burnout. Where relevant, we also aimed to describe any variability in outcomes by comparing counselors with varying degrees of experience in their counseling roles (newly trained, months of experience, and years of experience). Our overall goal was to use participant responses to inform strategies to improve counselor experiences, which should improve intervention delivery and quality. Because the intervention was designed with the goal of minimizing the added burden for lay providers (described below), it offers a helpful lens and case study for understanding the specific contribution of role shifts in relation to other stressors associated with lay counselor experiences.

## Methods

Semi-structured interviews and focus group discussions (FGDs) were conducted to explore lay counselor experiences providing a family therapy intervention in Eldoret, Kenya. Lay counselors were recruited from two pilot studies of the intervention in 2015 and 2017. Data collection occurred July–August 2018. All study procedures were approved by the ethical review boards at Duke University and Moi University in Kenya. Written informed consent was obtained for all activities.

### Setting and research team

The study was located in peri-urban communities surrounding the town of Eldoret, Kenya. Eldoret is located in the Rift Valley Province and is the fifth largest Kenyan city [29]. Some residents have access to mental health services through Moi Teaching and Referral Hospital, which provides limited inpatient and outpatient care. Available treatment focuses primarily on adults with serious mental illness and, to some extent, common mental disorders. Very little child- or family-specific training or treatment is available, and community-based approaches are uncommon. The research team was composed of two doctoral level clinical psychologists who are co-principal investigators (one US-based, one Kenyan-based), graduate students from the US, Kenyan masters-level psychologists, and Kenyan research assistants. Team members collaborated throughout the planning, data collection, and data analysis stages of the project.

### Study context: intervention and implementation

*Tuko Pamoja* (TP, “We are Together” in Kiswahili) is a family therapy intervention designed to promote family functioning and child or adolescent mental health for families with difficulties in relationships. Eligibility for TP is intentionally broad, with content designed to target problems related to family conflict, communication, and organization that occur alongside both internalizing and externalizing child symptoms. Related, TP is components-based, tailored to fit the needs of each family, and focuses on generating solutions to influence the family system. It is not time-limited, with families allowed to move at their own pace. A full description of TP is provided in Puffer et al. [30].

For implementation in the pilot studies, lay counselors were recruited through existing social structures, such as religious and community organizations, and chosen because they already naturally engaged in informal helping roles and had shown sustained interest in such activities. None of the counselors in the study had any previous training related to counseling or formal counseling experience; rather, people sought them out for advice, and they reported spending significant time providing advice and listening to people experiencing individual or family problems. At times, this was performed in the context of another helping role, such as pastor, youth group leader, or village community leader (often referred to as “policy maker”). Training in the family intervention was intended to augment their current, informal practices with evidence-based strategies. Counselors completed approximately 60 h of training. After being trained, counselors were expected to commit the same time to counseling as they dedicated to their advice-giving activities before they were trained, therefore not increasing their workload. This was important, as they were not paid for the counseling. The implementation approach was designed to be sustainable without external resources by integrating into *existing* volunteer activities and keeping counselors’ time commitment the same. However, the lay counselors were paid for their participation in research-related activities, such as surveys and interviews.

After training, counselors led the process of recruiting families to counsel, mirroring the natural ways they had already been connecting with families for informal counseling. In most cases, families had expressed a need to the counselor previously, but the counselor had not yet addressed the need in depth. The goal was to have counselors recruit from the types of families they were already helping so that the types and severity of problems would be representative of those they would have been expected to address in their day-to-day lives. Given this, problems ranged in complexity but were appropriate for the intervention and the counselors, especially with access to support and supervision. Counselors received supervision from local Kenyan supervisors after each session (in-person or via phone) that included feedback, planning for next steps, and brief refresher training and practicing skills as needed. Local supervisors received weekly consultation from clinical psychologists in Kenya and the United States [9].

The TP counseling sessions were held in the homes of the counseled families. In the first pilot trial of TP, counselors held an average of 15 sessions with a mean length of 40 min, totaling an average treatment exposure of 9 contact hours. Pilot results are promising for both family- and individual-level outcomes [30].

### Participants

Lay counselors were included in this study if they completed TP training as part of a pilot study. We categorized them into three groups based on level of supervised TP counseling experience: “Moderate” (n = 8), “Minimal” (n = 6), and “Training Only” (n = 6). The Moderate experience group included individuals who started TP counseling in 2015 and had counseled 2–3 families (Pilot Study 1), whereas the Minimal group started TP counseling in 2017 and had been assigned a single family (Pilot Study 2). The Training Only group were individuals from both pilot studies who had undergone training but had not yet provided TP for case-specific reasons, including changes in availability or difficulties engaging families. Of the 25 eligible counselors, 20 were available, and all 20 participated in this study. Of the remaining five, three had moved away, and two had outdated contact information.

Demographically, the three counselor experience groups were similar (see Additional file 1: Appendix S1 for a detailed demographics table). The overall mean age was 46, with individual group means ranging between 43 and 51. Of all the counselors, half were women, 16 were currently married, and 3 were widowed. All had some formal education: five had primary-level education (two did not complete primary); six had some secondary education; three completed secondary education; and five had some post-secondary education, with one having a university degree. Every counselor except one had a position, either formal or informal, that provided them income. This included four business owners, two casual workers, three policy makers, five farmers, three pastors, a school cook, and a village elder. All except one identified as Christian, who identified as Muslim.

### Data collection

A Kenyan research assistant completed all data collection activities following a 5-day training in study aims, data collection methods, and research ethics. Semistructured interviews (n = 20) were conducted in Kiswahili using a guide developed and piloted collaboratively by the Kenyan and US-based researchers. Questions focused on lay counselors’ approaches to counseling before TP training, changes in identity since becoming a TP counselor, and how TP counseling affected experiences of stress, burnout, motivation, and self-efficacy. For the Training Only group, questions related to formal counseling experiences were not included. The full interview guide is available in Additional file 1: Appendix S2. During interviews, participants completed a free-listing activity where they listed current sources of stress, not necessarily associated with the counselor role. The participant then ranked the items from most to least stressful, with the option to rank multiple items at the same level.

Interviews were audio recorded and transcribed from Kiswahili directly into English by a Kenyan research assistant fluent in both languages. As each transcript was completed, we conducted preliminary data analysis using close reading of transcripts, memoing, and preliminary code development. This process helped to make iterative adjustments to the interview guide, such as selecting a simpler Kiswahili translation or providing a clarifying metaphor. Participants were compensated for their time with 300 KSH (~ 3 USD).

After all participants from each counselor experience group completed their individual interviews, FGDs were conducted for the purpose of member checking—the process of presenting preliminary results to ask participants to provide feedback about whether the interpretations of their responses are accurate. It is also a chance to expand upon their responses or delve deeper into interpretations [31]. During each session, participants were presented with preliminary results from interviews with TP counselors from their group and asked to clarify, verify the information, and to expand on specific ideas that individuals mentioned. FGD participants also completed a pile sorting activity, a method used to understand how participants categorize items within a domain [32]. As a group, participants sorted index cards listing the stressors from free lists, placing them into piles with as few or as many stressors as deemed appropriate and labeling the stressor categories. The goal was to consolidate the numerous stressors into higher-level groups to assist with later stress mapping, described below. FGDs were conducted primarily in Kiswahili, with occasional English. They were audio recorded and transcribed directly into English by a Kenyan research assistant. Participants were compensated for their time with refreshments and 300 KSH (~ 3 USD).

### Analysis

Thematic content analysis was used to analyze the data [33]. All interview and FGD transcripts were first reviewed by the first author through close readings, where emergent themes were extracted and then reviewed with another researcher. Themes were organized into a codebook containing parent and child codes, code definitions, examples, and inclusion/exclusion criteria [34]. Most codes reflected research topics on the guide (e.g., identity, stress, burnout, motivation, and self-efficacy), whereas others emerged from the data (e.g., role mixing, burnout resistance). To identify problematic codes and issues with code application, we used an intercoder reliability exercise. Transcripts were independently coded one-by-one by two researchers. After each transcript, the researchers discussed discrepancies between coded segments and revised the codebook. This process was repeated until sufficient intercoder reliability was established (Kappa coefficient of 0.51 and overall agreement percentage of 94%). All transcripts were then coded by the first author.

Code summaries were developed to facilitate understanding and interpretation of themes. We determined the data to be thematically saturated, as the final four individual interview transcripts yielded no new themes. This was further supported by the fact that in FGDs, no new themes arose; participants only offered examples of previously-identified themes. Code summaries were then compared across counselor experience groups to check for patterned differences. Primarily, themes did not differ across groups; we indicate in results where they do differ. Finally, a concept diagram was devised from data interpretations to provide a visual description of study domain interactions.

A Stress Map was created for each experience group using the ranked lists of stressors from interviews and grouped categories from FGDs. The goal of this visualization analysis was to better understand the stressors that were most influential in counselors’ lives. Methods from Participatory Risk Mapping [35] were adapted to produce stress maps that visually display relative prevalence and severity of stressors. Such quantitative analyses of qualitative data are common with methods such as ranking and pile sorting, with results visually interpreted as reflecting relative rather than absolute values [36].

The prevalence index was the proportion of a group that listed a stressor category at least once. The prevalence index is therefore ordered between 0 and 1, with higher values representing stressors named by a higher proportion of group members. The severity index standardized each stressor rank within a participant’s list, where each stressor (i) and its rank (r) with total (n) risks identified by each participant (j) was calculated as ${\text{s}}_{\text{ji}} = 1 - \frac{{\left( {r_{i} - 1} \right)}}{{\left( {n_{j} - 1} \right)}}$. If an individual listed multiple items within a category (e.g., daily provisions and school fees within the poverty category), their severity scores were averaged. The severity index is therefore ordered between 0 and 1, with higher values representing stressors ranked as being more stressful. These individual severity values were then averaged across the group to produce a group severity index value for each stressor category. Values were plotted in four quadrants that represent above/below mean severity and prevalence to help visually identify key stressor clusters within and between groups.

## Results

### Role identities and shifts

To understand counselors’ personal expectations in their new formal counseling roles and their perceived counselor identity, participants were asked what they thought it means to be a TP counselor and to discuss key personal qualities of a TP counselor. Their developing sense of “professional identity” was important to understand because their new formal role and training were not officially announced to the community but only to the individual families they worked with. Across all groups, participants described a TP counselor as someone who is a good listener, empathetic, ready, flexible, has good communication skills, “takes their time,” and is a responsible leader. They noted specifically that a TP counselor differs from community elders in that TP counselors are not judgmental in their advice-giving; instead, they help families identify their own solutions. Finally, being a TP counselor was described as being a role model in the community because “nobody will love your work if your actions are not straight” (Male, 46, Moderate Group).

Participants described that before TP training, they offered advice and counsel anywhere they went: after church, on the streets, while running errands, or wherever they met someone in need. Their advice focused on encouraging or teaching from Bible scriptures, drawing from personal experiences, passing along wisdom from elders, praying with others, and giving direct advice on how to solve a specific problem. Recipients of their “advising,” as they often called it, were either people who sought their advice directly or those referred to them by someone else.

Consistent with role identity theory, when counselors began to integrate their new formal counselor role, they experienced some positive role shifts as well as tensions between roles. Some described that their new counseling skills generalized in ways that enhanced their performance in related professional or volunteer positions. For instance, “TP has affected me to some extent because I cannot use law enforcement like before; I no longer take people to the police so easily, and I try to solve the disputes” (Male, 46, Moderate Group). In some cases, counselors described this resulting in increased respect and appreciation from others. On a personal level, counselors described improvements in their roles within their families as they applied the TP skills to their own lives (described below).

The majority of TP counselors reported few problems with balancing roles because of the required responsibilities of the counselor role itself, suggesting the counseling role fit with their ongoing routines without significant interruptions. However, there were some cases where taking on the role of TP counselor sometimes conflicted with responsibilities associated with their other roles, especially when logistical challenges, like scheduling problems, required additional time. Counselors described “sacrificing time” to engage in counseling, leaving less time to fulfill their many other responsibilities. One pastor described that “one cannot really have time to prepare a sermon, visit members of the congregation, and have time for your own family” (Male, 58, Minimal Group). Though not mentioned frequently, one female counselor described making sure that counseling did not interfere with income-generating activities, saying, “when I am helping others, I should not forget about my own family… Since I am the sole provider for my children, if I don’t work, what will they eat?” (Female, 55, Moderate Group). Though the TP intervention was designed so that counselors were not spending more total time on counseling, the shift to meeting with the same families on a regular basis restructured time demands in ways that necessitated shifts in how they balanced multiple roles.

### Motivation

When asked why they became counselors, participants typically discussed intrinsic motivations first. As expected, every experience group mentioned a general desire to help others: “I was never happy at heart to see people languishing in their sorrows. I could never abandon or neglect them. I had to do something about it” (Male, 28, Training Only). Participants from each experience group also mentioned a deep-rooted calling from God and a passion for counseling and serving people after observing widespread community problems. The belief that they were making a difference and fulfilling God’s work was highly motivating. When families engaged in counseling, attended sessions, and showed positive changes or outcomes, the counselors were motivated to continue working.

Many expressed that receiving training improved motivation to counsel families because they had increased knowledge and new skills to handle a broader set of cases. This in turn increased their sense of self-efficacy and helped sustain them even through challenges or negative feedback. Participants also noted increased motivation after seeing the impact of applying some of the skills to their own lives, as described further below. Additionally, counselors reported receiving encouragement, respect, and empowerment from supervisors and fellow counselors, as well as from family and community members. These were especially helpful in maintaining motivation when client families were struggling, not progressing, or having problems scheduling sessions. One counselor noted, “I really loved the fact that I wasn’t left alone after the training… I got a lot of encouragement to continue” (Male, 63, Minimal Group).

### Self-efficacy

To explore self-efficacy, counselors were asked about their feelings of preparedness and perceived ability to meet expectations. The resounding narrative was that counselors felt empowered in their abilities to fulfill their counselor role after completing TP training. With this improved self-confidence and empowerment from training came a change in how counselors viewed their counselor role. When discussing their counseling approach before, many described their role as “shallow,” “reckless,” and having “no consistency.” Now, counselors felt they were more “professional” and were able to meet the expectations of their client families: “Before, we were practicing in the dark, unlike now, we are working in the light. TP has given us knowledge, equipped us, and widened our minds” (Female, 47, Moderate Group).

Despite overall improvements in self-efficacy, it was tested and often fluctuated based on client family trust, openness, and progress through therapy. Though they clearly experienced expanded counseling capacity, the counselors consistently requested that TP continue to provide more trainings. They expressed a persistent desire to improve their counseling abilities to handle an even wider breadth of problems.

### Stress

**Fig. 1:**
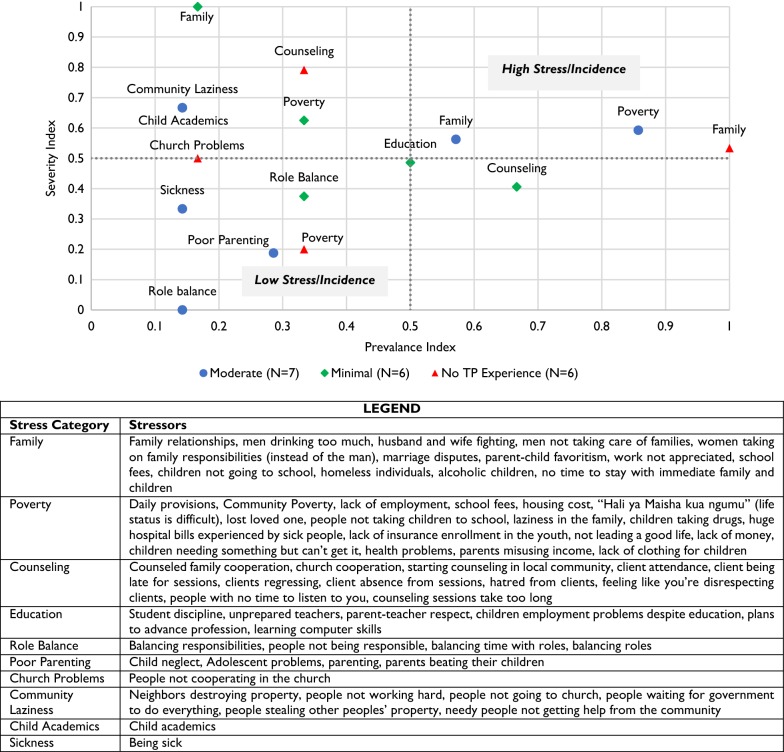
Counselor stress map by experience level (N = 19, every participant was included in the analysis except one from the Moderate group who did not rank their stressor list)

The stress map (Fig. 1) helped identify top stress areas for each counselor experience group. As described in the methods, a top stress area is one that has both high prevalence (proportion of group listing the stressor) and severity (rated more stressful overall).

For all counselor experience groups, a top stress category was poverty, describing both personal (e.g., acquiring daily provisions, paying school fees) and community (e.g., lack of employment, people not taking children to school) sources. Another top category across multiple groups was family, describing both personal (e.g., marriage disputes) and community sources (e.g., homeless individuals, substance abuse). A third category was counseling, describing challenges with being a TP counselor both logistically and emotionally. The specific prevalence and severity of these stressors were different between experience groups. The top stress areas for moderate experience counselors were poverty, family, and poor parenting. For minimal experience counselors, they were counseling, education, poverty, and role balance. For training only counselors, they were family, informal counseling (not TP), and poverty.

Although participants mentioned a broad range of stressors in free-listing, they focused on a smaller set during individual interviews, as questions aimed to understand counseling-related stressors in depth. Participants across groups expressed similar stressors related to therapy scheduling, family attendance and engagement, and perceptions of the family’s progress. Stress first came from becoming accustomed to their new formal counseling role and logistics, such as providing reports, recording sessions, and making follow-ups. Then, family attendance problems were stressful, as family members were sometimes busy with other priorities or avoided counseling when it became difficult. Counselors described arriving to empty, padlocked houses or wasting time waiting for family members to show up. At times, they questioned if they had done something wrong to cause a disinterest or avoidance of sessions.

During therapy sessions, counselors noted stress if they perceived lack of family engagement, which was essential for conducting the counseling. One counselor noted difficulty “getting them to open up; it takes a lot of time sometimes when none of them believed that their problems will remain confidential” (Female, 57, Moderate Group). One possibility raised by a counselor was research-specific activities (e.g., recordings and documents) might have made it harder for counselors to build rapport and trust. It was also difficult to maintain engagement and progress when families were undergoing acute hardships, which made it hard for them to focus on longer-term goals during the sessions. Examples included losing a job, not having enough money to buy food, or experiencing a medical emergency.

Participants also noted stress when trying to navigate counseling across gender and age differences, such as a woman counselor advising a man, a younger counselor counseling elders, or a counselor attempting to facilitate inter-generational communication in the family.

### Burnout

The interviewer used a “spring metaphor,” developed with the Kenyan team during interview guide revisions, to help describe experiences of burnout. Counselors were asked to think about any times they felt so compressed or stretched by their TP counseling work, they felt they could not continue working. Twelve of the 20 counselors endorsed feeling this way at times, including counselors from all experience groups. When discussing burnout, counselors often described instances where stress piled on continually without relief. This was most commonly discussed in connection to the scheduling and attendance stressors described previously. During times of burnout, counselors experienced pronounced negative thoughts or emotions related to these challenges that interfered with their drive to continue: “You find they are not there; then when you go there again, they tell you they are not ready for you; then it happens again…you feel like you are tired. That is what can make you lose hope” (Male, 47, Moderate Group). Additionally, some counselors experienced burnout when feeling like they could not meet the expectations of their supervisors, which led to hopelessness and the desire to avoid supervision—a required activity for counselors.

Participants were asked to reflect on how such situations made them feel in their head, heart, and body. All counselor groups expressed physical experiences of fatigue, pain, or feelings of sickness during such instances of overwhelming stress: “[It] makes you feel troubled. You lack peace. You feel even more tired as though you have been digging a shamba [field]” (Male, 47, Moderate Group). Counselors also expressed thoughts related to self-doubt and questioning of their capabilities as a counselor. At the core of the burnout experience was a struggle caused by the sincere desire to help—and belief that they had the skills to help—juxtaposed against families not engaging or making progress. In these situations, counselors noted “questioning myself” as they judged their counseling capabilities. Emotionally, participants described an overall lack of peace, loss of hope, and feeling “burdened in my heart.”

### Coping and supports

#### Coping with stress and burnout

Participants described similar coping mechanisms for dealing with both stress and burnout. For many counselors within all experience groups, stress relief came in the form of religious practices. These included praying, reading the Bible because it “makes the burden lighter,” and gospel music because “the songs have encouraging words” or “help me remove my mind from the issues” (Male, 47, Moderate Group). In addition, counselors invested in personal relationships, including talking to their family, spouse, or friends; playing with children; and “sharing ideas” with others.

Stress was also relieved by accessing support provided through TP, including information, supervisors, and peers. They described reviewing the therapy manual to reassure themselves they knew what they were doing, calling their supervisor, and meeting with other counselors. Some also mentioned that returning to their other roles outside of counseling was helpful as a coping strategy because such actions helped counselors distract and distance themselves from the situation. One counselor said, “They [other tasks] help me to remove my mind from the issues that the family [is] going through and not dwell on it” (Male, 47, Moderate Group). Some counselors also mentioned reframing stressors as a way to cope, alongside reminding themselves that they are likely temporary: “The whole world encounters diverse challenges and stresses; for me, I have decided to take it as a normal thing” (Female, 47, Moderate Group).

In instances of burnout, counselors tended to focus on techniques that renewed their motivation and distracted them. They mentioned reviewing training documents to reassure themselves they were implementing the counseling correctly, as well as their case notes, which often showed some progress had been made. These strategies often restored feelings of self-efficacy. Beyond these strategies counselors could perform, burnout also began to resolve if a family finally attended a session or showed progress; this often gave a sense of renewal and relief: “Now I am feeling okay. You know when you help a family and they get healed, you feel even much better” (Male, 47, Moderate Group). Even if such progress does not occur, counselors usually remained actively engaged in counseling despite feelings of burnout because of the strong convictions that led them to become a TP counselor or a strong sense of duty to TP: “I used to feel bad. I wasn’t happy; it was really hurting. Though it was something that I had decided that I would do, whether it be bad or good, I would push through to the end” (Male, 44, Minimal Group).

For the eight counselors who reported not experiencing burnout, we asked them why they thought they avoided the experience of overwhelming stress. The first protective factor was a strong sense of self-efficacy because of having gone through training, having their capacity widened, and being “built up” in counseling skills. This was connected to intrinsic confidence in one’s counseling capabilities. Some described a feeling of imperviousness to burnout, with one saying he “never felt that they [a family] could overwhelm me” (Male, 50, Moderate Group): “No, I didn’t feel like that [burnout]. We underwent thorough training where my knowledge and capacity was widened. I have learned how to handle a lot of issues” (Male, 46, Moderate Group).

A second protective factor was supervision, received in person or via phone. These counselors felt they could receive help when needed, and supervision made them feel part of a team and appreciated and recognized for their hard work: “We have all the resources we need to use during the process like the mobile phone. We are also given constant advice on challenges we face. The team is also doing good in checking on us through phone calls” (Male, 63, Minimal Group). A third protective factor was having a strict schedule for activities so that they could best manage their time. This helped the counselors better balance their multiple responsibilities and roles and perhaps even compartmentalize stress in a positive way: “I always try to ensure that I have a schedule of work so that I don’t overstretch myself… There are days I have set for counseling, and there are days that I have set for my own work.” (Female, 55, Moderate Group). A final protective factor was maintaining motivation based on the potential for overall improvement in a counselor’s community, even when dealing with stressors related to a specific family. They took pride in being part of making their community more welcoming and harmonious.

#### Personal skills application

All counselor groups described applying TP counseling concepts to their own lives. Some saw this as a necessary step for becoming role models and gaining credibility, and the changes they made contributed to positive role shifts on a personal level. Counselors most commonly reported changing the ways they interacted with their own families, including their behavior toward their spouses and how they disciplined their children. They described using the problem-solving processes and communication skills taught in TP, which they noticed helped them to control their reactions to negative emotions. Several counselors reported this led to positive relationship changes, such as increased love and togetherness in their home because of less quarreling and more open discussions, overall interactions, and time together. One counselor described reductions in marital conflict:

The training that I got from TP helped me reflect in my life and of my relation with my spouse whereby [in the past] we would quarrel at the top of our voices, not caring, disclosing family issues to the public. Our togetherness as a result of that change so far has helped us accomplish a lot. (Female, 47, Moderate Group)

Another counselor described applying his new counseling skills directly to his spouse and children:

I can say that I used to be very harsh and judgmental, giving final answers to issues; unlike now I know where I have come to learn that counseling is a process… now I can sit with my wife and children as a family and I listen to them so that I can be able to help them, because as a parent, I am also a counselor at home. (Male, 40, Moderate Group)

In addition to family improvements, a few counselors also noted becoming more approachable, getting along easier, and having stronger bonds within their church and community, and subsequently receiving increased respect, encouragement, and appreciation—again contributing to positive role shifts.

### Interactions among stress, burnout, motivation, and self-efficacy

**Fig. 2:**
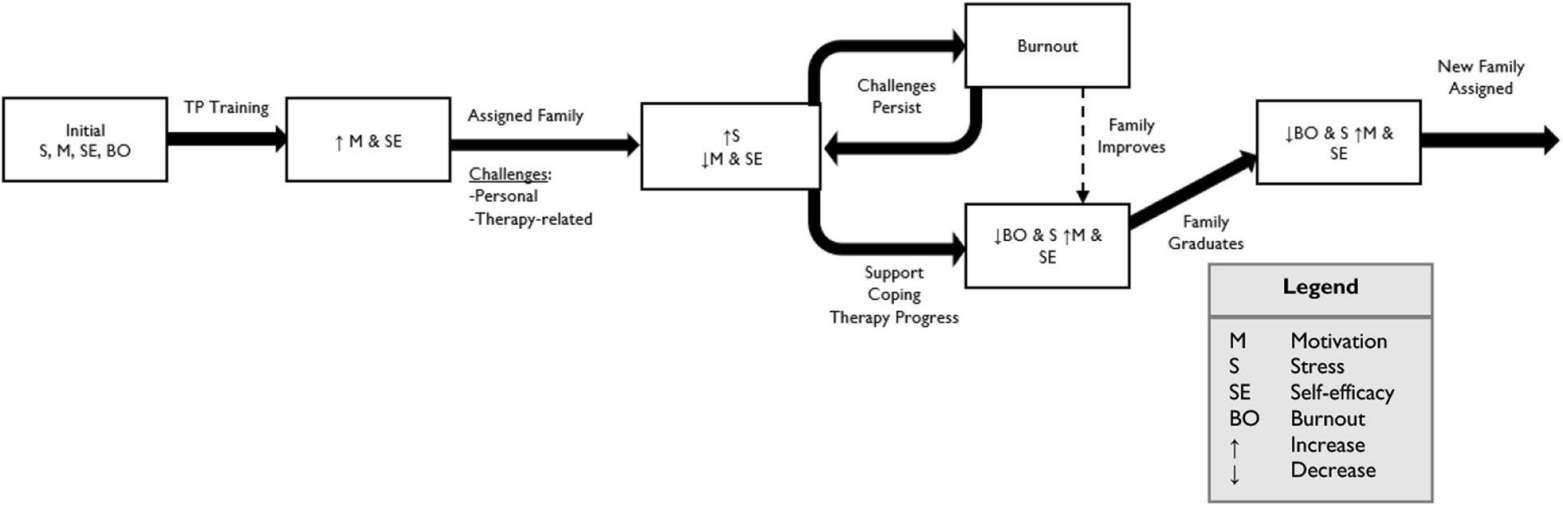
TP counselor changes in stress, burnout, motivation, and self-efficacy

We identified a common cycle the counselors reported experiencing over time (Fig. 2). Following TP training, counselors experience an increase in self-efficacy because of their improved counseling abilities. The TP skills and manual made them feel that they could be more systematic when helping families and that they had an expanded scope of situations with which they could help. This high self-efficacy was accompanied by an increase in motivation to counsel because of a sense of readiness and preparation. After being assigned a family, counselors experienced the first challenges of formal counseling despite their preparations and increased self-efficacy: personal challenges from balancing roles in trying to make time for counseling and therapy-related challenges of engagement and slow (or no) progress in early sessions. For some, these caused stress to increase while motivation and self-efficacy decreased.

With persistent challenges, the stress increased and persisted until a counselor could begin to experience burnout. This was driven by even further decreases in motivation and self-efficacy as a counselor questioned the commitment of the family they were counseling and their own capabilities. The struggles related to balancing roles as counselors with other responsibilities also continued, contributing to this negative cycle. When counselors had access to and engaged support and coping strategies, or the counseled family made progress, the negative effects of the challenges could be reduced. This included reduction in stress and burnout and an upswing in motivation and self-efficacy. Notably, sometimes this cycle repeated multiple times during the course of counseling for just one family.

Finally, in most cases, families graduated from counseling, having succeeded in reaching some or all of their counseling goals. At this point, both those who had experienced more support and coping, as well as those who had experienced more prolonged stress, often reported feeling a sense of accomplishment reflected in reduced feelings of stress and burnout and restoration of motivation and self-efficacy.

## Discussion

The purpose of this study was to conduct a humanistic examination of lay counselors’ experiences of role shifts, in the context of a family therapy intervention in Kenya. The lay counselors were already informally advising others in their communities, which provided a unique opportunity to examine changes due to a shift from an informal to formal counseling role. Taking on a new formal counseling role produced changes in the lay counselors’ lives due to their counseling duties and, more importantly, how their new counseling skills altered their approach to their other roles. This included changes in motivation, stress management techniques, and how they perceived their role in their community and families. Results highlight both the positive and negative experiences of lay counselors, and the ways in which these interact.

### Positive experiences

Similar to other lay provider populations, counselors expressed clear intrinsic reasons for becoming TP counselors [28], and they maintained those foundational motivations throughout their experience. This is not surprising, given that such strong motivational forces have been found in a number of studies [13, 14]. TP counselors saw their new role as an outlet to fulfill their intrinsic desires to help the community and achieve fulfillment. Most connected this intrinsic motivation explicitly to their religious beliefs and values related to helping others through their abilities as counselors that were now even stronger than before. This idea of using one’s talents to benefit others is one that is applicable across many religions and types of spirituality, and religiosity has been associated with more volunteering behaviors [37]. Further, those who perceive their work to be a calling from a higher power may be more involved and invested in their work [38]. This body of research is small, but available findings resonate with our results and point to the importance of acknowledging the role of faith and the potential benefits of partnering with faith-based organizations in task-shifting efforts.

TP counselors also applied the skills to their own lives, which they saw improve their relationships and ability to fulfill other roles, including professional and volunteer roles. This is perhaps related to the “helper therapy principle” described by Riesman [39, 40], whereby lay providers who share similar characteristics or problems of their clients receive “helper benefits.” These benefits might promote retention, motivation, and effectiveness of a range of lay provider-delivered interventions [41–43].

The new TP counselor role was manageable for most, perhaps because they were already engaged in informal counseling, showing a pre-existing interest in spending time on these activities. Unlike Mlotshaw et al.’s population of lay providers, who described multiple changes in identities and roles, TP counselors seemed to experience less of a role shift both in terms of identity change and adjusting to logistical requirements and maintenance of motivation [28]. This suggests the model of working with these “natural counselors” might have benefits over models in which lay providers are given multiple new responsibilities related to multiple health needs in which they might lack a specific interest [44]. In TP, the new roles also led to increased respect and status in the community for many counselors, which is also documented among lay providers in other studies, including those delivering home-based care in South Africa [44].

### Challenges

Although TP counselors seemed to experience less of a strained role shift due to their previous informal experience and the positive outcomes they described, the challenges with which they needed to cope are important and informative. First, although entering the role of a formal counselor was positive in many ways and seemingly less disruptive than with other delivery models, the new counseling role did, at times, compete with other roles. Some had to shift their schedules to guard against allowing the counseling role to interfere with their other responsibilities. Second, counselors were now going beyond providing informal advising to providing more consistent care for families experiencing multiple difficulties in relationships that were often accompanied by unstable living situations, and overall lack of organization in the home that made it difficult for them to keep agreed upon appointments. Although it is not surprising that this is a difficult population to engage in therapy, coping with attendance challenges can be defeating for counselors. Third, even when families engage, family therapy is, by its nature, a difficult process; conflict is often necessary, clients often experience resistance, and progress can be slow. These clinical challenges lead to stress as counselors cope with the uncertainty about whether families will improve. Although some of these stressors are likely unique, other studies have found that CHWs addressing a variety of health needs also have experienced stress related to caring for people with multiple complex needs that make delivery more difficult and lead to emotional distress, as they are unable to help with all of the presenting needs [45, 46].

Challenges experienced by counselors were associated clearly with experiences of stress and burnout that were particularly pronounced in the middle of the counseling process with a family, sandwiched between positive experiences. The initial motivation and high self-efficacy experienced at the beginning diminished as challenges persisted over time, leading over half of counselors to experience stress and some to experience burnout. Similar to other studies, counselors experienced physical manifestations of stress; they questioned their abilities; and they began to lose hope that the families would ever make progress, feeling that their efforts may be futile [45, 47]. Their stories emphasize the importance of not only these case-by-case scenarios that caused changes in stress or burnout but the mounting interactions between them.

When hitting these low points, counselors identified many effective coping strategies that led to a restoration of a positive perspective. Some utilized the behavioral coping skills taught in the therapy for emotion regulation; they reached out to their social networks for emotional support; they met with their counselor peers to provide mutual support; and they used supervision to gain both emotional support alongside problem-solving. More literature on these naturally-occurring coping efforts of lay counselors across contexts and interventions would help in developing interventions that build on existing strengths.

### Implications/recommendations

Integrating counselor supports more intentionally and explicitly within interventions could buffer lay counselors’ stress and enhance the benefits of taking on these types of roles. We recommend that lay provider trainings and supervision have more formalized components that discuss potential challenges they might face, allowing them to understand the role more fully and to consider together how to prepare. Trainings should then include explicit activities to help counselors prepare for and buffer future stressors, such as forecasting and troubleshooting expected challenges ahead of time, normalizing expected negative emotions, and teaching and practicing coping strategies or problem-solving skills to prevent burnout—including the same skills that are included as part of the intervention itself. Counselors in this study certainly applied skills from the intervention to their own lives, and this process could be formalized and facilitated by trainers and supervisors. In ongoing supervision, this process of support in coping and problem-solving should continue, providing structured, frequent opportunities for lay counselors to share their own emotional responses and difficulties that arise throughout the process. This process of implementation support is recognized as an important component of supervision in the training of mental health professionals in high-resource settings [48]. Because supervision efforts are already difficult to scale [49, 50], this can be a brief check-in to provide ongoing support with a mechanism for flagging concerns.

Additionally, connecting with peers can provide opportunities for mutual support during role transitions, stress, and burnout in a flexible and low-cost way. Interventionists should foster a collaborative atmosphere among their workforce and coordinate peer-support strategies. This can be through formal mechanisms, such as in-person meetings and/or through telecommunication for virtual meetings or casual communication (e.g., WhatsApp groups). In one TP pilot study, counselors organically developed peer support groups, citing these as important spaces for coping [9]. In other programs, peer support is likely happening within the context of peer supervision, though their support of one another has not been closely studied. An example of a method to mobilize this organic peer support process was adopted in a study in Myanmar through providing workshops for trauma management medics to learn techniques to promote personal and peer mental wellbeing [51]. By providing lay providers with both the tools and collaborative spaces to work through their issues, such as with peer support groups or targeted workshops, they can become more proactive in dealing with problems that may arise in their work.

Future task-shifting interventions should carefully consider the demands placed on lay providers related to time, effort, and stress and identify ways to balance those demands with appropriate compensation or incentives. The WHO has recognized this need in their CHW guidelines [49] as important for promoting high motivation and retention, improving counselor experience, and recognizing that the needs and rights of providers are important in all task-shifting efforts. In our implementation model, the strategy is to lower demand and burden, with a maximum caseload of two families, and to integrate responsibilities into roles counselors had prior to TP training (e.g., informal counselors within their communities). This was not a perfect solution, as counselors met challenges such as requiring additional transport and frustration with participant attendance; at times, counseling demands interrupted their work-related activities, exceeding the anticipated burden. They benefitted from small additional incentives to continue, similar to other settings where training opportunities, certificates, and ID cards serve as small, additional benefits [8, 52]. Even when intended demand on lay providers is relatively low, it is essential to carefully consider demand and value in developing a compensation plan, ideally in collaboration with lay provider input. Monetary compensation should always be provided in cases where high workload prohibits the lay provider from meeting family financial demands through other employment avenues [13, 18]. If financial resources are not available, it is essential to reduce demand and burden.

### Limitations and future directions

One specific limitation related to analysis is that we coded the transcripts in English after translation from Kiswahili, though the inclusion of member checking with FGDs allowed opportunities to get feedback to clarify any questions about translations or interpretations. Further, although it is a strength of this study that almost all of the eligible counselors participated, findings are limited by the fact that their experience was related to one specific intervention and geographical location. Future work will be valuable in understanding the similarities and differences across contexts, types of mental health interventions, and more diverse groups of counselors. Of particular interest is how socioeconomic status of counselors may influence levels of stress and burnout during non-compensated counseling activities—even with implementation strategies designed to minimize burden. In addition, while difference across counselors of different experience groups did not emerge across most domains in this study, future work should examine how counselors’ experiences change over time. Developing and evaluating lay counselor support strategies is also a clear future direction, as this is the essential step for improving outcomes and minimizing negative consequences for lay providers playing such an integral role in filling the global mental health treatment gap.

## Conclusion

As global health initiatives continue to involve lay providers, it is important to understand their experiences so they can be adequately supported. Counselors in this study reported both positive and challenging aspects of their new roles, with most experiencing initial high motivation and self-efficacy that waned amidst challenges in providing treatment, creating stress and periods of burnout. They also reported effective coping strategies that restored motivation and self-efficacy on which future intervention approaches can build. Task-shifting initiatives might improve lay provider experiences and ultimate success of the interventions themselves by providing more formal, intentional support structures.

## Supplementary information

**Additional file 1: Appendix S1.** TP Counselor Demographics. **Appendix S2.** Individual Interview Guide.

## Data Availability

The datasets used and/or analyzed during the current study are available from the corresponding author on reasonable request

## Abbreviations

LMIC: Low and middle income countries
CHW(s): Community health worker(s)
FGDs: Focus group discussions
TP: Tuko Pamoja
