# Supplementary material for: What about lay counselors’ experiences of task-shifting mental health interventions? Example from a family-based intervention in Kenya
Source: Int J Ment Health Syst. 2020 Feb 20;14:9. doi: 10.1186/s13033-020-00343-0 (PMC7031864; doi:10.1186/s13033-020-00343-0)
Supplement: Supplementary file 1 — Additional file 1: Appendix S1. TP Counselor Demographics. Appendix S2. Individual Interview Guide. [file 13033_2020_343_MOESM1_ESM.docx]

**Appendix S1. TP Counselor Demographics**

TP Counselor Demographics by Experience Group

|  | **Experience Group** | | |
| --- | --- | --- | --- |
|  | **Moderate** | **Minimal** | **Training Only** |
| Size | 8 | 6 | 6 |
| Gender (male) | 50% | 50% | 50% |
| Age Range (Mean) | 35-57 (47) | 43-63 (51) | 28-53 (43) |
| Education |  |  |  |
| Unreported | -- | -- | 1 |
| Some Primary | 2 | 1 | 2 |
| Some Secondary | 5 | 2 | 2 |
| Diploma^a^ | 1 | 2 | 1 |
| University | -- | 1 | -- |
| Occupation |  |  |  |
| Community Leader^b^ | 2 | -- | -- |
| Church Leader^c^ | 5 | 3 | 4 |
| Farmer | -- | 1 | -- |
| Business | -- | 1 | -- |
| Police | 1 | -- | 2 |
| Unemployed | -- | 1^d^ | -- |

^a^_Lower than a University degree_

^b^_Policymaker, Village Elder_

^c^_Pastor, Sunday School Teacher_

^d^_Participant had some coursework and training in counseling, but no work experience_

**Appendix S2. Individual Interview Guide**

******************************************************************************

# ***BEFORE STARTING***

**“Before starting, I would like to emphasize that we are not sharing any of your responses with your supervisor or anyone on the Tuko Pamoja team. Any information that is shared will be not be associated with you. Your responses will remain confidential and will not stop you from continuing to be a Tuko Pamoja Counselor”**

******************************************************************************

# “Now, I am going to ask a couple simple questions to help us start our discussion.”

1. How long have you been counseling as a Tuko Pamoja counselor?
2. What do you like most about being a Tuko Pamoja counselor?

# “Now, I would like to change the conversation towards our first topic. For the next few questions, think back to the time before you started training with Tuko Pamoja.”

1. Before becoming a counselor for Tuko Pamoja, what was your role in the community?
   1. What was your role in the church, your family?
2. What caused you to take on this role in your community?
3. Can you tell me a little bit about your counseling approach before Tuko Pamoja?
   1. Where did you learn these techniques, advices, etc.?
4. When you were approached about becoming a Tuko Pamoja counselor, how did you decide to do it?

a. What was your personal goal for your position as a counselor before Tuko Pamjo?

# “Thank you so much for your responses so far. Now, I would like to ask you about your time as a counselor since you started using your training from Tuko Pamoja.”

1. How many families have you counseled with the Tuko Pamoja program?
2. What does it mean to you to be a Tuko Pamoja counselor?
   1. What are the qualities of being a Tuko Pamoja counselor?
   2. If misunderstood: How have you started to use the Tuko Pamoja training skills in your counseling sessions?
3. Now that you are a counselor for Tuko Pamoja, how has your role in the community changed?
   1. In what ways is it different from when you were counseling before Tuko Pamoja training?
   2. What do other people in the community think is your role?

# “Thank you for your responses. Now, when people take on new responsibilities, sometimes it can also change the way they feel and think about themselves, families, friends, or other things. I’d like to talk about anything that’s happened like that for you.”

1. In what ways has your counseling role changed your day-to-day life? [**Community, family, spouse, etc.**]
   1. Can you give an example of this change?
2. Have your relationships with other people changed since becoming a Tuko Pamoja counselor? **[i.e. family members, spouse, church members]**
   1. In what ways have they changed in a **good** way?
   2. In what ways have they changed in a **bad** way?
   3. Do people treat you differently now that you are a Tuko Pamoja counselor?
      1. In what ways do they treat you differently?

# “Before continuing, I would like to again emphasize that we are not sharing any of your responses with your supervisor. Your responses will remain confidential and have no bearing on you continuing to be a Tuko Pamoja Counselor”

**“Now, I would like to talk about your stressful experiences while being a Tuko Pamoja counselor, and how these experiences made you feel.”**

1. What are some things that are stressful about being a Tuko Pamoja counselor?
   1. What makes these things stressful? [**Why are they stressful?**]
   2. What’s the hardest thing about being a Tuko Pamoja counselor?
2. Do you feel more stress in life since becoming a Tuko Pamoja counselor [**completed training**]?
   1. In what ways has your stress changed? Can you give an example?

# [If they say they are not more stressed even though they listed a lot of stressors, ask how that works or how is that possible]

- - - 1. [**Relist the stressors to them in your probe**]

1. What are ways that you help reduce or relieve your stress?
   1. How effective [**helpful, useful**] are those?
   2. What else might be helpful for relieving stress?
      1. Maybe something you haven’t tried to do?
2. How is your relationship with your supervisor? [**Emphasize confidentiality here**]
   1. Who was your supervisor? [**Helping them remember**]
   2. How is your communication with them?
   3. Are there ways they could better support you?
3. How is your relationship with other Tuko Pamoja counselors?
   1. Similar probes to previous question

# “Thank you so much for your responses so far. Now, the next section is similar to the last, but this time, I will be asking about your experiences with larger, extreme, or overwhelming feelings of stress related to your tasks as a Tuko Pamoja counselor. Imagine a time when you were counseling a family as a Tuko Pamoja counselor, and you felt like a candle that had been all but used up; a time when you felt burnt out like a candle from stress and working so much.”

1. What brought on these feelings of overwhelming stress?
   1. Do you ever feel like you have too many responsibilities or are taking on more than you initially thought you would? [**Do you have an example?**]
2. Can you describe how this situation made you feel?
   1. Be specific on how these situations:
      1. Make you feel in your heart?
      2. What are you thinking about in your head?
      3. Make you feel in your body?
      4. Other areas?
3. How do you relieve these feelings of overwhelming stress?

# [If they say “nothing” or “don’t know”, ask about community and support relationships]

1. Although you have these feelings of overwhelming stress, what keeps you motivated to continue being a Tuko Pamoja counselor?
   1. Are there things your supervisor or others in Tuko Pamoja could do to help relieve these feelings of stress or [**insert vocabulary participant has used**]?

# “Now, this final section will be related to topics of your feelings about your preparation and training to become a Tuko Pamoja counselor, and how capable you feel as a Tuko Pamoja counselor”

1. At the start of becoming a Tuko Pamoja counselor, did you feel you were prepared to take on these responsibilities?
   1. Has this feeling changed since then, now that you have some experience?
2. Were the trainings helpful in preparing you for your responsibilities as a Tuko Pamoja counselor?
   1. In what ways were they helpful? Ways trainings were unhelpful?
   2. What did you learn from the trainings compared to what you used to do?
3. What are your own personal expectations for yourself in your role as a Tuko Pamoja counselor?
   1. [**If not understood**] What is your goal for being a Tuko Pamoja counselor?
   2. Do you meet these personal expectations?
      1. In what ways do [**or don’t**] you meet them?
4. What are the expectations others, like the families you counsel, your family, your spouse, your community, place on you as a Tuko Pamoja counselor?
   1. Do you feel you are able to meet their expectations?
      1. In what ways do [**or don’t**] you meet them?

# Closing Questions: Never Skip these

1. If you met someone who was considering becoming a Tuko Pamoja counselor, how would you describe the responsibilities to them?
   1. What advice would you give them?
      1. Advice on making the decision?
      2. Advice on performing the responsibilities?
2. Are there ways in which you think Tuko Pamoja could better support future counselors?
   1. How would such things help Tuko Pamoja counselors with their day-to-day tasks?
3. Was there anything that you would like to mention or discuss that was not mentioned so far in our conversation?
4. Demographic Questions
   1. Age, Gender/Sex, Home/Village of Origin, Primary Church location, Number of families counseled?

**Closing**: “Thank you so much for your time and participation in this interview. Your time is very much appreciated and your insights have been helpful.”

*****************************************************************************
